# Supplementary material for: Single missense mutations in Vi capsule synthesis genes confer hypervirulence to Salmonella Typhi
Source: Nat Commun. 2024 Jun 19;15:5258. doi: 10.1038/s41467-024-49590-6 (PMC11187135; doi:10.1038/s41467-024-49590-6)
Supplement: Supplementary file 1 — Supplementary Information [file 41467_2024_49590_MOESM1_ESM.pdf]

Supplementary Information

Single missense mutations in Vi capsule synthesis genes confer hypervirulence to *Salmonella* Typhi

Gi Young Lee[1] and Jeongmin Song[1, *]
[1] Department of Microbiology and Immunology, Cornell University College of Veterinary Medicine, Ithaca, New York 14853

*Correspondence and requests for materials should be addressed to Jeongmin Song (jeongmin.song@cornell.edu)

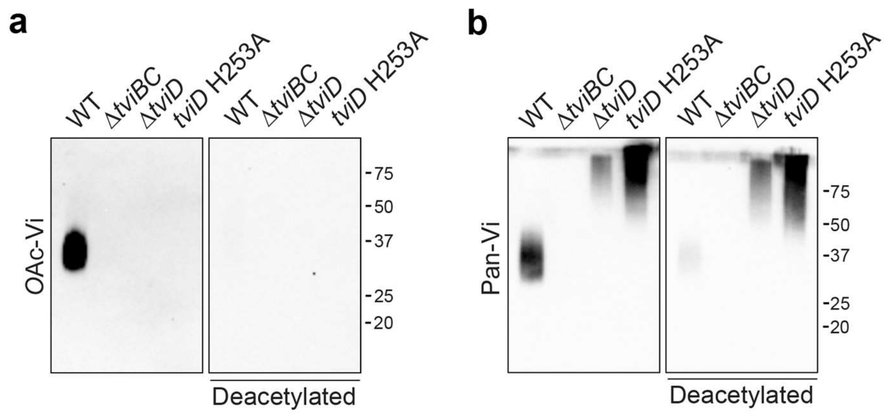

Supplementary Figure 1, related to Figs. 1-4. Validations of two Vi antibodies used in this study. Antibodies detecting 3-*O*-acetylated Vi (OAc-Vi) and total Vi (detecting both unmodified and 3-*O*-acetylated Vi, Pan-Vi) have been validated. Δ, deletion. *tviD* H253A, a catalytic-inactive mutant. Deacetylated, hydroxylamine (NH_2OH)-mediated deacetylation. When indicated, the membrane was incubated with PBS/0.5 M NH_2OH for 2 hours.

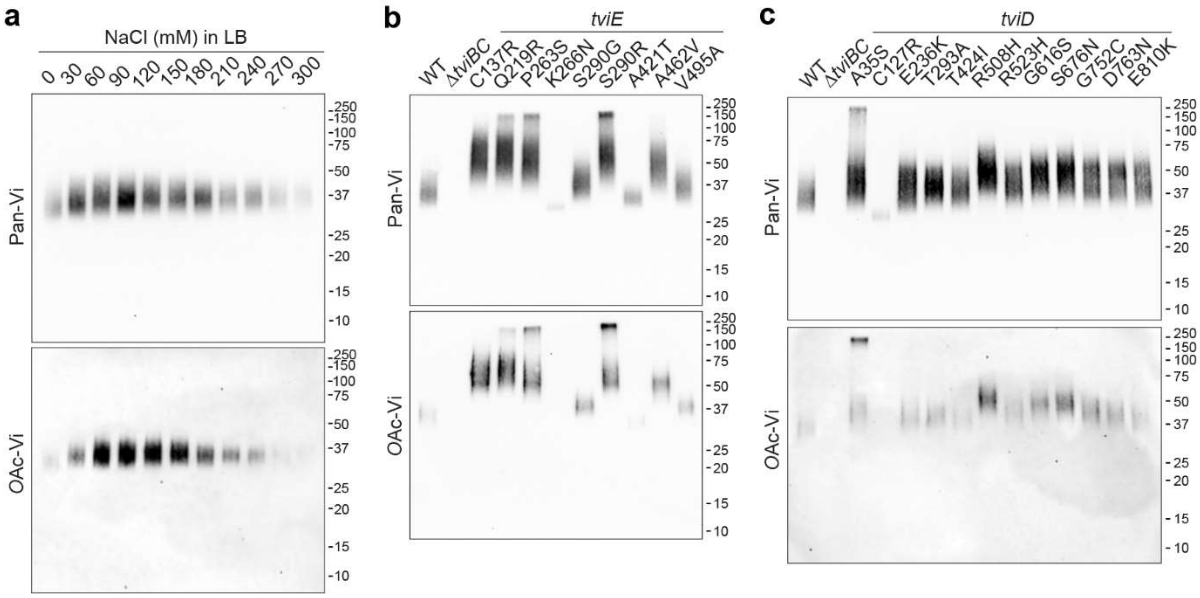

Supplementary Figure 2. related to Fig. 1e and 1g. Untrimmed immunoblot results shown in Fig. 1e (**a**) and 1g (**b** and **c**).

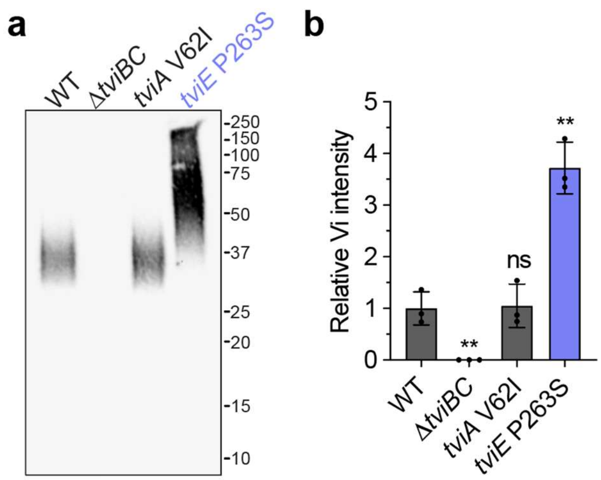

Supplementary Figure 3. related to Fig. 1g. *tviA* V62I is not a phenotype-changing mutation. A representative immunoblot result (**a**) and quantification of 3 experiments (**b**). Two-tailed t-tests between WT and indicated strain were performed. Bars represent the mean ± SD.

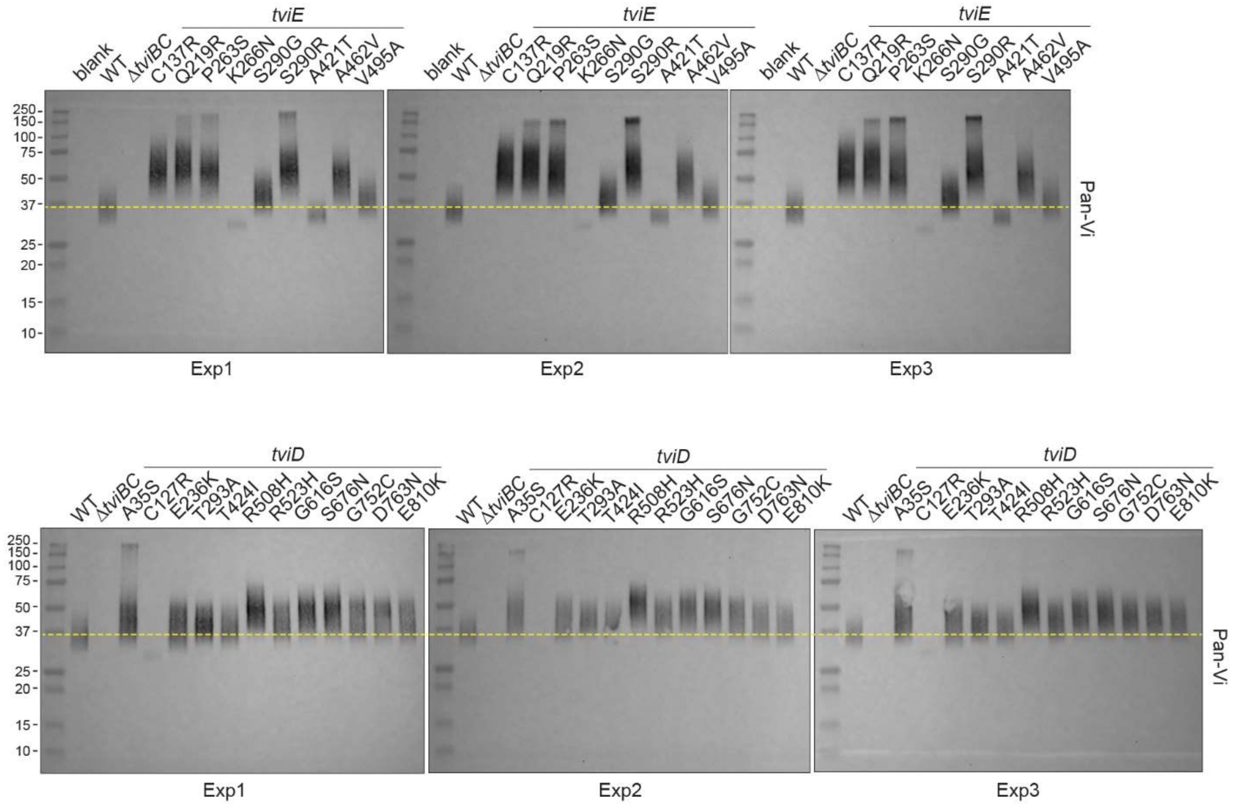

Supplementary Figure 4, related to Fig. 1g-k. Immunoblot results of three independent experiments. Yellow horizontal dotted lines are added to indicate the decreased or increased Vi length compared to WT Vi.

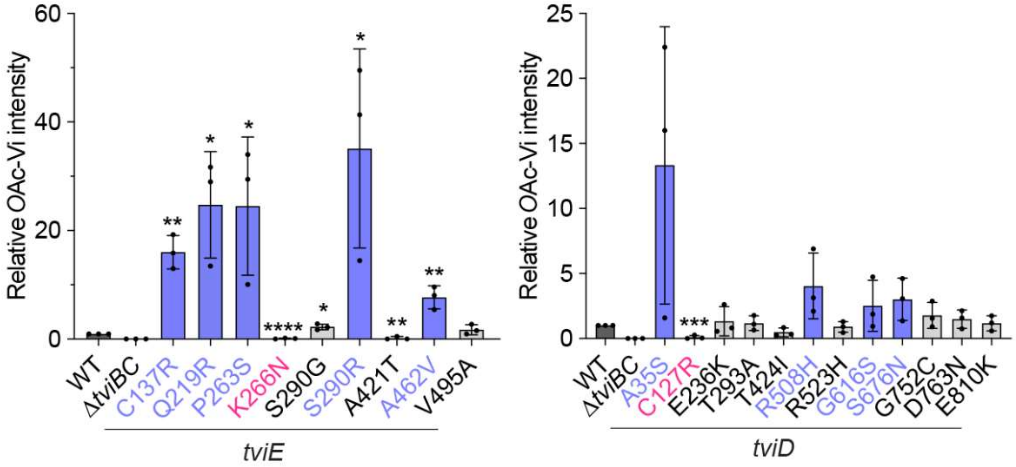

Supplementary Figure 5, related to Fig. 1g. Relative acetylation intensity of *S.* Typhi WT and Vi variants. Two-tailed t-tests between WT and indicated strain were performed. Bars represent the mean ± SD.

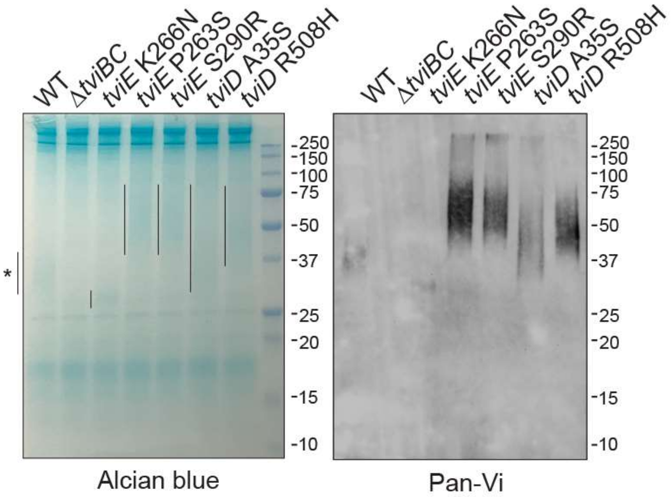

Supplementary Figure 6, related to Fig. 1g. Alcian blue-SDS-PAGE (left) and immunoblot (right) methods for the detection of *Salmonella* Vi. One-half of the cultures of the indicated *S.* Typhi strains were analyzed by alcian blue SDS-PAGE, while the remaining half of the samples were analyzed using immunoblots simultaneously. The molecular weight differences are similar in both results. However, there are numerous non-specific background signals present in alcian blue-stained SDS-PAGE, in contrast to the specific detection of Vi observed in immunoblot results. Vertical lines in the alcian blue SDS-PAGE are for WT Vi, hypo Vi, and hyper Vi.

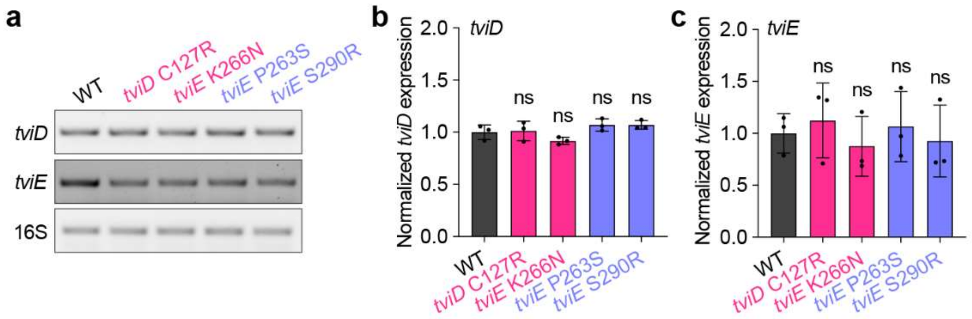

Supplementary Figure 7, related to Fig. 1g. The WT and Vi variants demonstrate similar levels of expression for the *tviE* and *tviD* gene products. a-c, RT-qPCR results (**a**) and quantification of 3 experiments (**b-c**). Primers used are listed in S. Table 5. Two-tailed t-tests between WT and indicated strain were performed. Bars represent the mean ± SD.

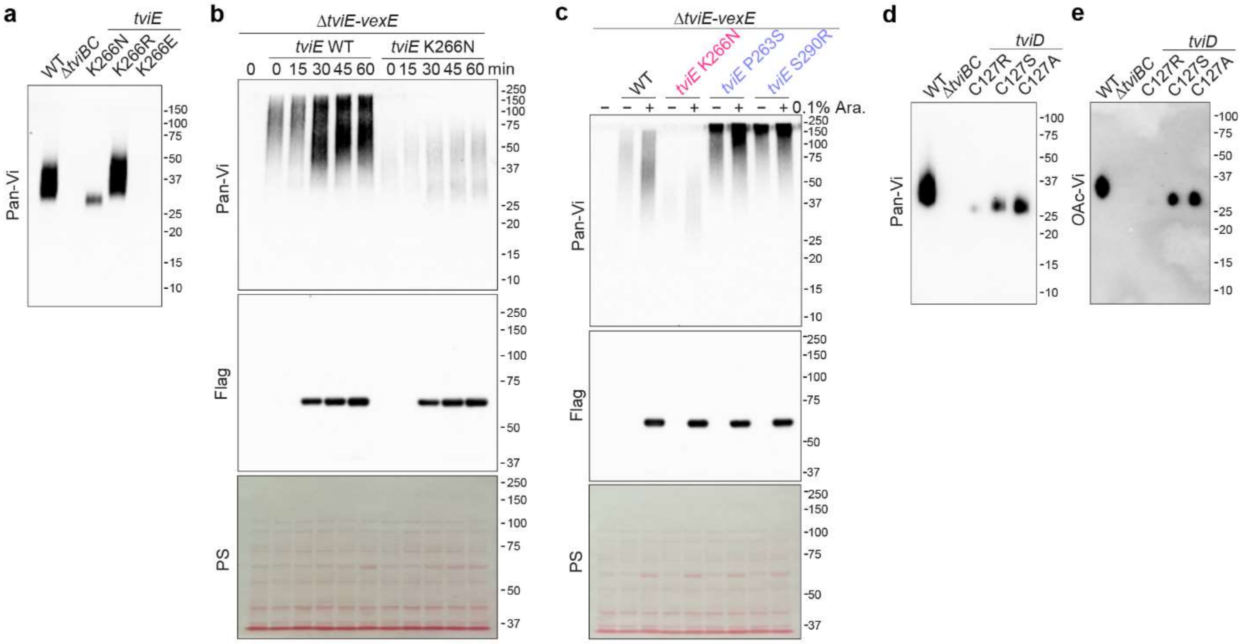

Supplementary Figure 8, related to Fig. 2. Untrimmed immunoblot results shown in Fig. 2e (**a**), 2g (**b**), 2h (**c**), 2l (**d**), and 2m (**e**). PS, Ponceau S stained membrane**.**

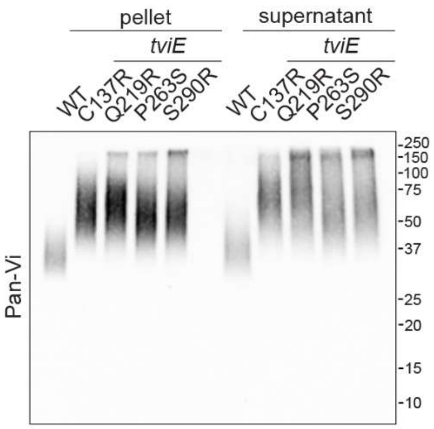

Supplementary Figure 9, related to Fig. 4i. Untrimmed immunoblot results shown in Fig. 4i.

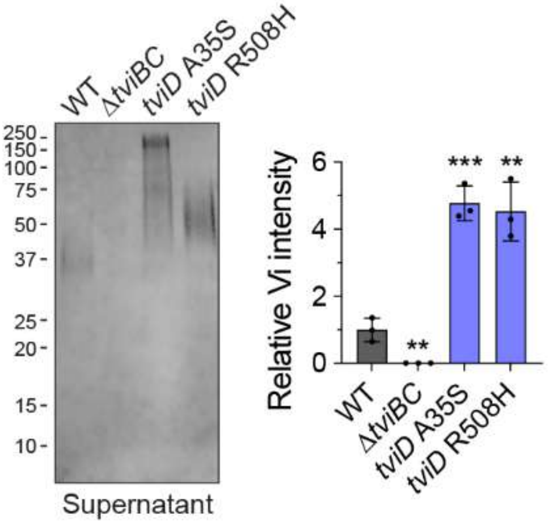

Supplementary Figure 10, related to Fig. 4i-j. Immunoblots assessing the shedding of Vi by *S.* Typhi WT and *tviD* mutants. Representative immunoblot result (left) and quantification of three immunoblots (right). Two-tailed t-tests between WT and indicated strain were performed. Bars represent the mean ± SD.

Supplementary Table 1, related to Fig. 1d. List of clinical missense mutations characterized in this study.

Gene	Mutation	Counts	Total strains
tviE	C137R	27	5,256
	Q219R	68	
	P263S	102	
	K266N	87	
	S290G/R	148/46	
	A421T	35	
	A462V	31	
	V495A	38	
tviD	A35S	50	5,257
	C127R	42	
	E236K	102	
	T293A	33	
	T424I	52	
	R508H	68	
	R523H	36	
	G616S	31	
	S676N	41	
	G752C	28	
	D763N	46	
	E810K/G	19/14	

Supplementary Table 2, related to Fig. 1g-k. Mucoidy test results.

Group	Strain	Blood agar plate	LB agar plate
WT	WT	Negative	Negative
Acapsule	Δ*tviBC*	Negative	Negative
Hyper Vi capsule	*tviD* A35S	Negative	Negative
	tviD R508H	Negative	Negative
	tviD G616S	Negative	Negative
	tviD S676N	Negative	Negative
	tviE C137R	Negative	Negative
	tviE Q219R	Negative	Negative
	tviE P263S	Negative	Negative
	tviE S290R	Negative	Negative

Supplementary Table 3, related to Fig. 4a-d. Summary of percent survival and SHIRPA.

**Strain**	Challenge dose (CFU/mouse)	Challenge route	Survivors/Total (% survival)	Body condition (well-conditioned)	Trunk curl (present)
WT	8 x 10^5	IP	9/9 (100%)	9/9 (100%)	0/9 (0%)
tviE K266N	8 x 10^5	IP	6/6 (100%)	6/6 (100%)	0/6 (0%)
tviE P263S	8 x 10^5	IP	5/10 (50%)	0/10 (0%)	9/10 (90%)

**Strain**	Touch escape (moderate)	Coat appearance (well-groomed)	Eye discharge (present)	Diarrhea (present)	Liver abscesses (present)
WT	9/9 (100%)	9/9 (100%)	0/9 (0%)	0/9 (0%)	0/9 (0%)
tviE K266N	6/6 (100%)	6/6 (100%)	0/6 (0%)	0/6 (0%)	0/6 (0%)
tviE P263S	0/10 (0%)	0/10 (0%)	5/10 (50%)	5/10 (50%)	7/10 (70%)

All dying mice showed eye discharge, diarrhea, and liver abscesses.
Eye discharge, diarrhea, and liver abscesses are not part of SHIRPA.
The occurrence of diarrhea may be higher than 50% since there is a chance that we missed diarrhea symptoms from the recovered and survived mice due to our 2 times daily monitoring.

Supplementary Table 4, related to Methods. List of bacterial strains used in this study.

ID	Description	
SB2201	*S*. Typhi ISP2825 WT	Wild type
JS0014	*S*. Typhi ISP2825 Δ*tviBC*	This study
JS0030	*S*. Typhi ISP2825 Δ*tviD*	This study
JS0031	*S*. Typhi ISP2825 Δ*tviE-vexE*	This study
JS0032	*S*. Typhi ISP2825 *tviD* A35S	This study
JS0033	*S*. Typhi ISP2825 *tviD* C127R	This study
JS0034	*S*. Typhi ISP2825 *tviD* E236K	This study
JS0035	*S*. Typhi ISP2825 *tviD* H253A	This study
JS0036	*S*. Typhi ISP2825 *tviD* T293A	This study
JS0037	*S*. Typhi ISP2825 *tviD* T424I	This study
JS0038	*S*. Typhi ISP2825 *tviD* R508H	This study
JS0039	*S*. Typhi ISP2825 *tviD* R523H	This study
JS0040	*S*. Typhi ISP2825 *tviD* G616S	This study
JS0041	*S*. Typhi ISP2825 *tviD* S676N	This study
JS0042	*S*. Typhi ISP2825 *tviD* G752C	This study
JS0043	*S*. Typhi ISP2825 *tviD* D763N	This study
JS0044	*S*. Typhi ISP2825 *tviD* E810K	This study
JS0045	*S*. Typhi ISP2825 *tviE* C137R	This study
JS0046	*S*. Typhi ISP2825 *tviE* Q219R	This study
JS0047	*S*. Typhi ISP2825 *tviE* P263S	This study
JS0048	*S*. Typhi ISP2825 *tviE* K266N	This study
JS0049	*S*. Typhi ISP2825 *tviE* K266R	This study
JS0050	*S*. Typhi ISP2825 *tviE* K266E	This study
JS0051	*S*. Typhi ISP2825 *tviE* S290G	This study
JS0052	*S*. Typhi ISP2825 *tviE* S290R	This study
JS0053	*S*. Typhi ISP2825 *tviE* A421T	This study
JS0054	*S*. Typhi ISP2825 *tviE* A462V	This study
JS0055	*S*. Typhi ISP2825 *tviE* V495A	This study
JS0057	JS0031 carrying pBAD-*tviE*-Flag plasmid (pJS0232)	This study
JS0058	JS0031 carrying pBAD-*tviE* K266N-Flag plasmid (pJS0233)	This study
JS0059	JS0031 carrying pBAD-*tviE* P263S-Flag plasmid (pJS0234)	This study
JS0060	JS0031 carrying pBAD-*tviE* S290R-Flag plasmid (pJS0235)	This study
JS0061	*S*. Typhi ISP2825 *tviA* V62I	This study
JS0063	*S*. Typhi ISP2825 *tviD* C127S	This study
JS0064	*S*. Typhi ISP2825 *tviD* C127A	This study
JS0065	*S*. Typhi ISP2825 *tviE* WT (Cm^R)	This study
JS0066	*S*. Typhi ISP2825 *tviE* P263S (Kan^R)	This study
JS0067	*S*. Typhi ISP2825 *tviE* K266N (Kan^R)	This study

Supplementary Table 5, related to Methods and S. Fig. 7. List of RT-qPCR primers used in this study.

**Semi-RT-qPCR primers**		
**Name**	**Sequence**	**Product size**
tviD_F	cagccgatgctttacagctgtttgataagt	221 bp
tviD_R	actgaatttgattgccaaagataa	
tviE_F	cttccaggttgtatataacggcgtctta	290 bp
tviE_R	cgaatagtattctctccagaatcc	
16S_F	gaaatgcgtagagatctggagg	146 bp
16S_R	cacaacctccaagtagacatcg	
