# Supplementary material for: Single missense mutations in Vi capsule synthesis genes confer hypervirulence to Salmonella Typhi
Source: Nat Commun. 2024 Jun 19;15:5258. doi: 10.1038/s41467-024-49590-6 (PMC11187135; doi:10.1038/s41467-024-49590-6)
Supplement: Supplementary file 3 — Description of Additional Supplementary Files [file 41467_2024_49590_MOESM3_ESM.pdf]

Additional Supplementary Data Files

Single missense mutations in Vi capsule synthesis genes confer hypervirulence to *Salmonella* Typhi

Gi Young Lee[1] and Jeongmin Song[1, *]
[1] Department of Microbiology and Immunology, Cornell University College of Veterinary Medicine, Ithaca, New York 14853

*Correspondence and requests for materials should be addressed to Jeongmin Song (jeongmin.song@cornell.edu)

Supplementary Data 1, related to Fig. 1a. Bioinformatic analysis results of 5,379 *S.* Typhi clinical isolates.
Data is attached as a separate Excel file.

Supplementary Data 2, related to Fig. 1a. Summary table for the Vi biosynthesis system of 5,379 *S.* Typhi clinical isolates.
Data is attached as a separate Excel file.

Supplementary Data 3, related to Fig. 1c. Summary of clinical missense mutations that occurred in the Vi biosynthesis system.
Data is attached as a separate Excel file.

Supplementary Data 4, related to Fig. 5e. Global distribution of *S.* Typhi capsular variants.
Data is attached as a separate Excel file.
