# Supplementary material for: Single missense mutations in Vi capsule synthesis genes confer hypervirulence to Salmonella Typhi
Source: Nat Commun. 2024 Jun 19;15:5258. doi: 10.1038/s41467-024-49590-6 (PMC11187135; doi:10.1038/s41467-024-49590-6)
Supplement: Supplementary file 8 — Reporting Summary [file 41467_2024_49590_MOESM8_ESM.pdf]

nature portfolio

Corresponding author(s): Jeongmin Song
Last updated by author(s): 2024-5-26

Reporting Summary

Nature Portfolio wishes to improve the reproducibility of the work that we publish. This form provides structure for consistency and transparency in reporting. For further information on Nature Portfolio policies, see our Editorial Policies and the Editorial Policy Checklist.

Statistics

For all statistical analyses, confirm that the following items are present in the figure legend, table legend, main text, or Methods section.

n/a	Confirmed	
☐	☒	The exact sample size (*n*) for each experimental group/condition, given as a discrete number and unit of measurement
☐	☒	A statement on whether measurements were taken from distinct samples or whether the same sample was measured repeatedly
☐	☒	The statistical test(s) used AND whether they are one- or two-sided<br>*Only common tests should be described solely by name; describe more complex techniques in the Methods section.*
☒	☐	A description of all covariates tested
☒	☐	A description of any assumptions or corrections, such as tests of normality and adjustment for multiple comparisons
☐	☒	A full description of the statistical parameters including central tendency (e.g. means) or other basic estimates (e.g. regression coefficient) AND variation (e.g. standard deviation) or associated estimates of uncertainty (e.g. confidence intervals)
☐	☒	For null hypothesis testing, the test statistic (e.g. *F*, *t*, *r*) with confidence intervals, effect sizes, degrees of freedom and *P* value noted<br>*Give P values as exact values whenever suitable.*
☒	☐	For Bayesian analysis, information on the choice of priors and Markov chain Monte Carlo settings
☒	☐	For hierarchical and complex designs, identification of the appropriate level for tests and full reporting of outcomes
☒	☐	Estimates of effect sizes (e.g. Cohen's *d*, Pearson's *r*), indicating how they were calculated

Our web collection on statistics for biologists contains articles on many of the points above.

Software and code

Policy information about availability of computer code

Data collection	All S. Typhi WGSs that were available in the NCBI's GenBank database (5,749 strains as of 11/03/21) were re-annotated using the Prokaryotic Genome Annotation Pipeline (PGAP, 2021-07-01.build5508; RRID: SCR_021329) to synchronize all the annotations under same criteria. Resulting data are included as S. Table 1.
Data analysis	PGAP, SeqSero2, Clustal Omega, and custom code was used to analyze over 5,000 S. Typhi WGS in this study. Resulting data are included as S. Table 1.

For manuscripts utilizing custom algorithms or software that are central to the research but not yet described in published literature, software must be made available to editors and reviewers. We strongly encourage code deposition in a community repository (e.g. GitHub). See the Nature Portfolio guidelines for submitting code & software for further information.

Data

Policy information about availability of data

All manuscripts must include a data availability statement. This statement should provide the following information, where applicable:

- Accession codes, unique identifiers, or web links for publicly available datasets
- A description of any restrictions on data availability
- For clinical datasets or third party data, please ensure that the statement adheres to our policy

The published article includes all datasets generated during this study. Source data are provided with this paper.

Research involving human participants, their data, or biological material

Policy information about studies with human participants or human data. See also policy information about sex, gender (identity/presentation), and sexual orientation and race, ethnicity and racism.

Reporting on sex and gender	Both males and females aged 18–65 years donated their blood samples; however, we did not record their biological sex information to conduct this study in a de-identified manner.
Reporting on race, ethnicity, or other socially relevant groupings	Blood donors who volunteered to donate ~10 ml of their peripheral blood were white or Asian.
Population characteristics	Blood donors were Ithacan adults.
Recruitment	Blood donors were recruited via email, in-person invitations, and word of mouth.
Ethics oversight	The process of collecting human blood samples was carried out in accordance with the protocol that has been approved by the Institutional Review Board for Human Participants at Cornell University (protocol number IRB0008664). A nurse practitioner at the Cornell Human Metabolic Research Unit conducted a peripheral blood draw to obtain primary neutrophils and sera. The data were analyzed in an anonymous manner. All adult participants (18-65 years old) provided informed consent. The written consent form was provided to all participants.

Note that full information on the approval of the study protocol must also be provided in the manuscript.

Field-specific reporting

Please select the one below that is the best fit for your research. If you are not sure, read the appropriate sections before making your selection.

☒ Life sciences ☐ Behavioural & social sciences ☐ Ecological, evolutionary & environmental sciences

For a reference copy of the document with all sections, see nature.com/documents/nr-reporting-summary-flat.pdf

Life sciences study design

All studies must disclose on these points even when the disclosure is negative.

Sample size	At least 3 independent experiments were performed throughout the study. We determined the sample sizes for mouse experiments using comparable animal studies that achieved statistical significance, which aligns with the sample sizes determined by power analysis.
Data exclusions	No data are excluded.
Replication	At least 3 independent experiments were performed throughout the study.
Randomization	We randomly divided the mice into the indicated groups, each of which received a different bacteria preparation, along with Vi preparations when indicated.
Blinding	Blinding was not used in this study integrating bioinformatic analysis of over 5,000 WGS, generation of a point mutation(s), characterization of those mutants, and structure-function studies. We present all data obtained, and at least 3 independent experiments were conducted throughout the study.

Reporting for specific materials, systems and methods

We require information from authors about some types of materials, experimental systems and methods used in many studies. Here, indicate whether each material, system or method listed is relevant to your study. If you are not sure if a list item applies to your research, read the appropriate section before selecting a response.

Materials & experimental systems

n/a	Involved in the study
☐	☒ Antibodies
☐	☒ Eukaryotic cell lines
☒	☐ Palaeontology and archaeology
☐	☒ Animals and other organisms
☒	☐ Clinical data
☒	☐ Dual use research of concern
☒	☐ Plants

Methods

n/a	Involved in the study
☒	☐ ChIP-seq
☒	☐ Flow cytometry
☒	☐ MRI-based neuroimaging

Antibodies

Antibodies used: Methods include detailed information including company name, catalog #, and lot #.

Validation: We conducted rigorous validation experiments of key antibodies of this study, which is provided as S. Fig. 1

Eukaryotic cell lines

Policy information about cell lines and Sex and Gender in Research

Cell line source(s): Henle-407 cells were obtained from the Galan laboratory and maintained in the Song laboratory.

Authentication: We frequently checked the cells for their morphological features, growth speed, and functionalities, focusing on their susceptibility and response to Salmonella and Salmonella toxins. We note that these responses were consistent throughout the entire study period for both the current research and the PI's prior research.

Mycoplasma contamination: The cell line used in this study was tested negative for mycoplasma.

Commonly misidentified lines (See ICLAC register): NA

Animals and other research organisms

Policy information about studies involving animals; ARRIVE guidelines recommended for reporting animal research, and Sex and Gender in Research

Laboratory animals: Cmah null mice were obtained from the Jackson Laboratory , maintained in the ECRF, and used for infection studies in the TMCF animal facilities at Cornell. All infection experiments were conducted in the TMCF animal facility (room C1029), which is a clean SPF ABSL-2 environment. This room in the TMCF follows a 10-14 light/dark cycle. The lights turn on at 5 a.m. and off at 7 p.m. The temperature range is 65-75 °F (18-24 °C), with humidity levels ranging from 30 to 70%. Food and water were provided ad libitum.

Wild animals: This study did not involve wild animals.

Reporting on sex: Both male and female mice were used. No sex-associated differences were observed.

Field-collected samples: This study did not involve samples collected from the field.

Ethics oversight: All mouse experiments were conducted following protocols approved by the Cornell University's institutional Animal Care and Use Committee (IACUC; protocol # 2014-0084)

Note that full information on the approval of the study protocol must also be provided in the manuscript.

Plants

Seed stocks: *Report on the source of all seed stocks or other plant material used. If applicable, state the seed stock centre and catalogue number. If plant specimens were collected from the field, describe the collection location, date and sampling procedures.*

Novel plant genotypes: *Describe the methods by which all novel plant genotypes were produced. This includes those generated by transgenic approaches, gene editing, chemical/radiation-based mutagenesis and hybridization. For transgenic lines, describe the transformation method, the number of independent lines analyzed and the generation upon which experiments were performed. For gene-edited lines, describe the editor used, the endogenous sequence targeted for editing, the targeting guide RNA sequence (if applicable) and how the editor was applied.*

Authentication: *Describe any authentication procedures for each seed stock used or novel genotype generated. Describe any experiments used to assess the effect of a mutation and, where applicable, how potential secondary effects (e.g. second site T-DNA insertions, mosiacism, off-target gene editing) were examined.*
